# Resveratrol Ameliorates Intestinal Damage Challenged With Deoxynivalenol Through Mitophagy *in vitro* and *in vivo*

**DOI:** 10.3389/fvets.2021.807301

**Published:** 2022-01-13

**Authors:** Yujian Huang, Changbing Zheng, Bo Song, Li Wang, Hao Xiao, Zongyong Jiang

**Affiliations:** ^1^State Key Laboratory of Livestock and Poultry Breeding, Ministry of Agriculture Key Laboratory of Animal Nutrition and Feed Science in South China, Guangdong Public Laboratory of Animal Breeding and Nutrition, Guangdong Provincial Key Laboratory of Animal Breeding and Nutrition, Maoming Branch, Guangdong Laboratory for Lingnan Modern Agriculture, Institute of Animal Science, Guangdong Academy of Agricultural Sciences, Guangzhou, China; ^2^Guangdong Provincial Key Laboratory of Animal Nutrition Control, Institute of Subtropical Animal Nutrition and Feed, College of Animal Science, South China Agricultural University, Guangzhou, China

**Keywords:** piglets, resveratrol, deoxynivalenol, mitophagy, intestinal function

## Abstract

Deoxynivalenol (DON) reduces growth performance and damage intestinal function, and resveratrol (RES) has positive effects on growth performance and intestinal function. The purpose of this study was to investigate the protective mechanism of RES *in vitro* and *vivo* challenged with DON. The results showed that dietary supplementation with DON significantly increase the mRNA expression levels of mitophagy- related genes, and protein level for PINK1, Parkin, Beclin-1, Lamp, Atg5, Map1lc, Bnip3, Fundc1, Bcl2l1 and SQSTMS1 (*P* < 0.05), while supplementation with both RES and DON decreased those indexes in the ileum. Besides DON significantly decreased protein level for Pyruvate Dehydrogenase, Cytochrome c, MFN1, OPA1, and PHB1 (*P* < 0.05), while supplementation with both RES and DON increased protein level for PHB1, SDHA, and VDAC in the ileum. Moreover, *in vitro*, we found that DON significantly decreased mitochondrial respiration (*P* < 0.05), while RES + DON increased the rate of spare respiratory capacity. Also, DON significantly decreased total NAD and ATP (*P* < 0.05), while RES + DON increased the total NAD and ATP. These results indicate that RES may ameliorates the intestinal damage challenged with deoxynivalenol through mitophagy in weaning piglets.

## Introduction

Deoxynivalenol (DON) or vomiting toxin, is the most common trichothecenes toxin produced by Fusarium, which mainly contaminates cereal crops (1). DON could reduce animal feed intake, vomiting, fever, diarrhea, and anorexia, even death. However, different animals have different sensitivity to DON. Pigs are the most sensitive animals among monogastric animals and ruminants (2–5). Recent study has shown that DON could reduce the growth performance of pigs, affect immune system, antioxidant system, cell signal transduction, gene expression and protein synthesis of livestock (6). Our previous studies have found that DON decreased the growth performance weaned piglets, destroyed intestinal function and structural integrity, weakened antioxidant capacity and protein synthesis levels of weaning piglets (2, 3). The mechanism by which DON exerts its toxicological effects is through binding to the phthaloyltransferase on the 60 s subunit of eukaryotic ribosomes, causing MAPK phosphorylation, inducing inflammatory process in the organism, and leading to lipid peroxidation damage in cell membranes, which in turn inhibition of protein and genetic material synthesis. However, the traditional chemical detoxification of DON pollution is unable to meet the low-energy, high-efficiency, and green environmental protection in modern farming. In recent years, the control of piglet stress damage through nutrition has received ever greater attention. Therefore, it is one of the hot spots in animal nutrition research to effectively mitigate DON damage to piglets through nutritional regulation to improve piglet growth performance.

Studies have shown that the addition of arginine, glutamic acid and antibacterial peptides to feed can effectively mitigate the toxic effect of DON on the intestinal damage in weaned piglets (4). RES is a bioactive material, naturally occurring polyphenolic plant antitoxin with anti-inflammatory, anti-aging, anti-cancer and cardioprotective properties. Found mainly in wine, blueberries, peanuts and other nuts, it is a potential additive to mitigate the toxic effects of DON (7). RES was shown to improve mitochondrial respiratory metabolism and lipid oxidation through Sirt1, and to target mitochondria to ameliorate stress damage (8). Mitochondria are the main target organelles for oxidative damage. Excessive ROS produced by damaged mitochondria will activate proteins such as p53 and Caspase to initiate apoptosis. Effective identification and removal of damaged mitochondria from the cells is therefore essential to ameliorate stress damage. Study shows RES attenuates the oxidative damage via mitochondrial autophagy in Parkinson's patients (9), and mitigate mitochondrial damage, and improve the intestinal function in diquat-challenge piglets (10). Thus, RES could mitigate organismal damage through mitochondrial autophagy, however, its mitigating effect on DON-induced intestinal damage are not well understood.

Therefore, in this study, by using DON-induced model *in vitro* and *vivo*, we want to know the protective regulatory role of resveratrol on intestinal function challenged with DON. This study provides a theoretical basis for the nutritional regulation of early weaned piglets.

## Materials and Methods

### Animals and Diets

All animal procedures used in the present study were approved by the Animal Care and Use Committee of Guangdong Academy of Agricultural Sciences and followed the Guidelines for the Care and Use of Animals for Research and Teaching. A total of 64 weaned piglets [Duroc × (Landrace × Yorkshire), 21 days old, barrow] with an initial weaning weight of 6.97 ± 0.10 kg were randomly allocated to four dietary treatments. The piglets fed a basal diet were considered the control group (CON), and the other groups were fed the basal diet supplemented with 300 mg RES/kg diet (RES), 3.8 mg DON/kg diet (DON) or 3.8 mg DON plus 300 mg RES per kg diet (DON+RES group) for a 28-days feeding trial. RES (> 99.0%) was obtained commercially from Shaanxi Ciyuan Biotechnology Co., Ltd. (Xian, China). Each treatment consisted of eight replicate pens, with two piglets per pen (n = 16 piglets per treatment). The basal diet was formulated to meet the nutrient recommendations of the National Research Council (NRC) 2012.

### Sample Collection and Processing

At the end of the experiment, eight piglets from each group were anesthetized and bled, the abdominal cavity was quickly dissected and the viscera removed. The intestinal segments were ligated and 1 cm sections of intestine were taken from the middle of the whole jejunum and ileum respectively, washed with pre-cooled PBS, collected in centrifuge tubes and snap frozen in liquid nitrogen, then stored at −80°C for further study.

### Cell Culture and Treatment

The cell culture refers to our previous study (11). High-glucose (25 mM) Dulbecco's modified Eagle's (DMEM-H), fetal bovine serum (FBS), and antibiotics were procured from Invitrogen (Grand Island, NY, USA). Plastic culture plates were manufactured by Corning Inc. (Corning, NY, USA). Unless indicated, all other chemicals were purchased from Sigma-Aldrich (St. Louis, MO, USA). IPEC-J2 cells were seeded and cultured with DMEM-H medium containing 10% FBS, 5mM l-glutamine, 100 U/mL penicillin, and 100 μg/ml streptomycin at 37°C in a 5% CO_2_ incubator. After an overnight incubation, the cells were changed to culture in 15 μmol/L RES for 24 h and then exposed to 0.5 μmol/L DON for another 24 h. Cells were treated or collected for the analysis of extracellular flux, and GC-MS.

### Real-Time PCR

The protocol of total RNA extraction, quantification, cDNA synthesis and real-time PCR was adapted from the method of (12). Briefly, total RNA was isolated from intestinal samples by using the Trizol method. Real time PCR was carried out by using forward and reverse primers (Supplementary Table 1) to amplify the target genes. For quantification, amplification efficiencies curves were constructed from serial 1:2 dilutions, and the 2^−ΔΔCT^ method was used to calculate the mRNA expression of the target genes relative to housekeeping gene (β-actin).

### Western Blotting Analysis

Frozen intestinal samples were collected as described by Tan et al. (13). Protein concentrations of tissue homogenates were measured by using the BCA method and bovine serum albumin as standard. All samples were adjusted to an equal concentration (50 μg protein). The western blotting was conducted based on previous description. The primary antibodies are LC-3B (1: 1,000; Cell Signaling Technology), P62 (1: 1,000; Cell Signaling Technology), Parkin (1: 1,000; Cell Signaling Technology), BNIP3/Nix (1: 1,000; Cell Signaling Technology), BNIP3 (1: 1,000; Cell Signaling Technology), Pyruvate Dehydrogen (1: 1,000; Cell Signaling Technology), COX IV(1: 1,000; Cell Signaling Technology), Cytochrome c(1: 1,000; Cell Signaling Technology), HSP 60(1: 1,000; Cell Signaling Technology), Mitofusin 1(1: 1,000; Cell Signaling Technology), Mitofusin 2(1: 1,000; Cell Signaling Technology), OPA1(1: 1,000; Cell Signaling Technology), PHB1(1: 1,000; Cell Signaling Technology), SDHA(1: 1,000; Cell Signaling Technology), SOD1(1: 1,000; Cell Signaling Technology), TOM20(1: 1,000; Cell Signaling Technology), VDAC(1: 1,000; Cell Signaling Technology) or β-actin(1: 1,000; Cell Signaling Technology). All protein measurements were normalized to β-actin.

### Extracellular Flux Assays

The XF-24 Extracellular Flux Analyzer and Cell Mito Stress Test Kit from Seahorse Biosciences were used to examine the effects of addition of different treated with 0 (NC) or 0.5 μmol/L or 1 μmol/L DON and 0 or 15 μM RES, respectively on mitochondrial respiration in IPEC-J2 cells. Cells in four replicates per group. Owing to the effects of DON on IPEC-J2 cell proliferation, total cellular protein was determined and used to normalize mitochondrial respiration rates.

### Gene Knockout With CRISPR-Cas9

Thanks to Yulong Yin lab for providing ATG5 plasmid (13). Guide RNAs were designed using the online CRISPR design tool (http://crispr.mit.edu/) and then cloned into the BbsI-digested plasmids (pSpCas9n) containing the entire guide RNA scaffold. The genomic region flanking the ATG5 target site was amplified using polymerase chain reaction (PCR). The products underwent a reannealing process to facilitate heteroduplex formation. After re-annealing, the products were treated with T7 Endonuclease I (NEB) following the manufacturer's recommended protocol. Then used lentiviral transfection, the viral solution was added to the cell culture medium and co-incubated with the cells.

### Statistical Analysis

Results are expressed as Mean ± SEM. The statistical analysis was performed by one-way ANOVA using SPSS 17.0 (SPSS Inc., Chicago, IL, USA). Probability values <0.05 and <0.01 were considered statistically significant. *P*-values were calculated using a two-tailed paired Student's *t*-test.

## Results

### Dietary Supplementation With RES Alleviated the Negative Effects on mRNA Expression Levels of Mitophagy-Related Genes Challenged With DON

**Figure 1:**
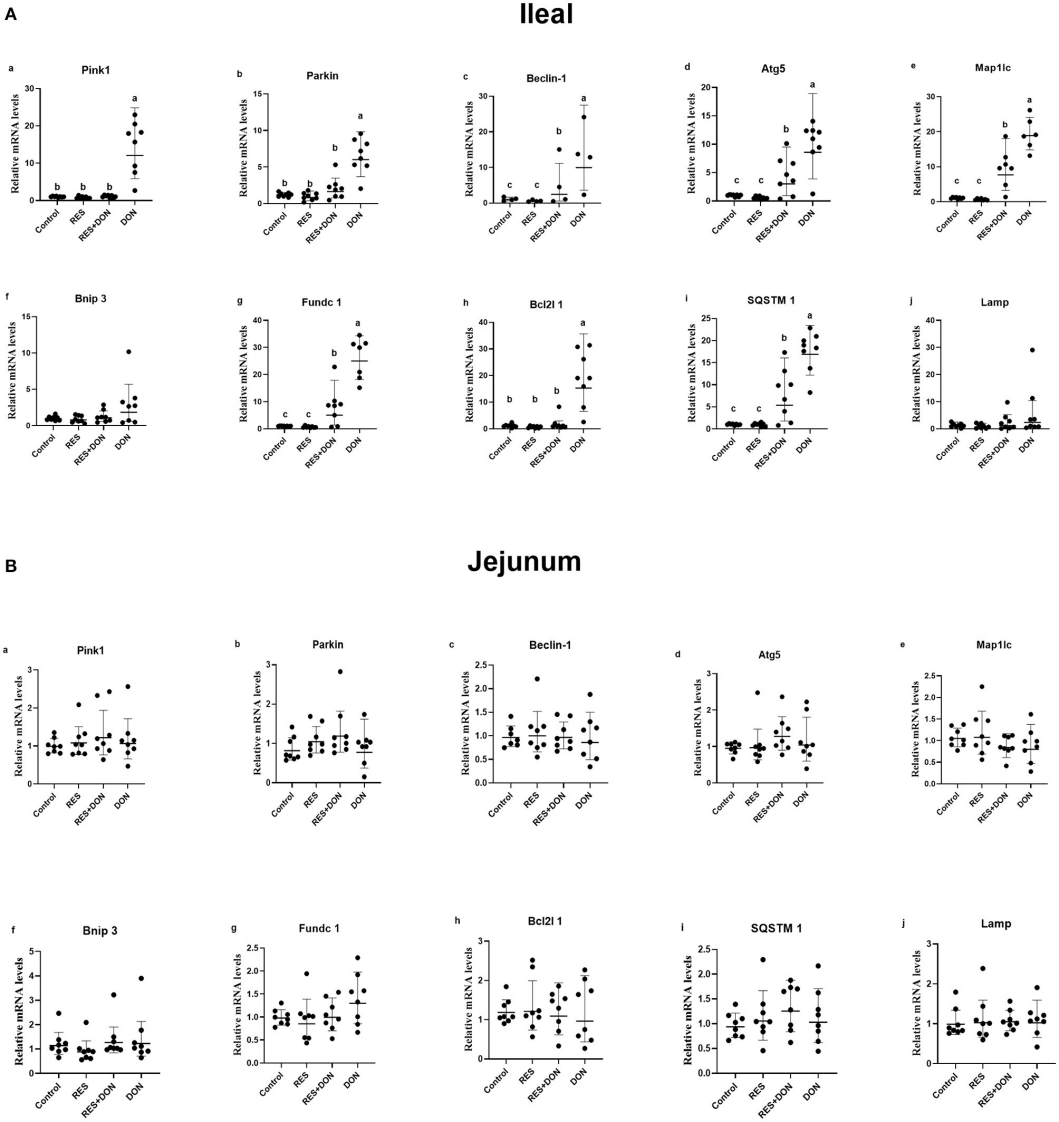
Dietary supplementation with RES alleviated the negative effects on mRNA expression levels of mitophagy-related genes challenged with DON. Data were expressed as means ± SEM of at least three independent experiments. **(A,B)** Values with different letters are significantly different (*P* < 0.05).

To determine the molecular mechanism of RES on DON-fed piglets, we analyzed the mRNA expressions of mitophagy-related genes in the ileum and jejunum of weaning piglets (Figure 1). Dietary supplementation with DON increased (*P* < 0.05) the mRNA expressions of PINK1, Parkin, Beclin-1, Lamp, Atg5, Map1lc, Bnip3, Fundc1, Bcl2l1 and SQSTM1 in the ileum, while there were no differences (*P* > 0.05) in those indexes among the Control, RES, and RES+ DON treatments in the ileum. However, there were no differences (*P* > 0.05) in the mRNA expressions of PINK1, Parkin, Beclin-1, Lamp, Atg5, Map1lc, Bnip3, Fundc1, Bcl2l1 and SQSTM1 expressions among the four treatments in the jejunum.

### Dietary Supplementation With RES Increases the Expression of Mitophagy-Related Genes

**Figure 2:**
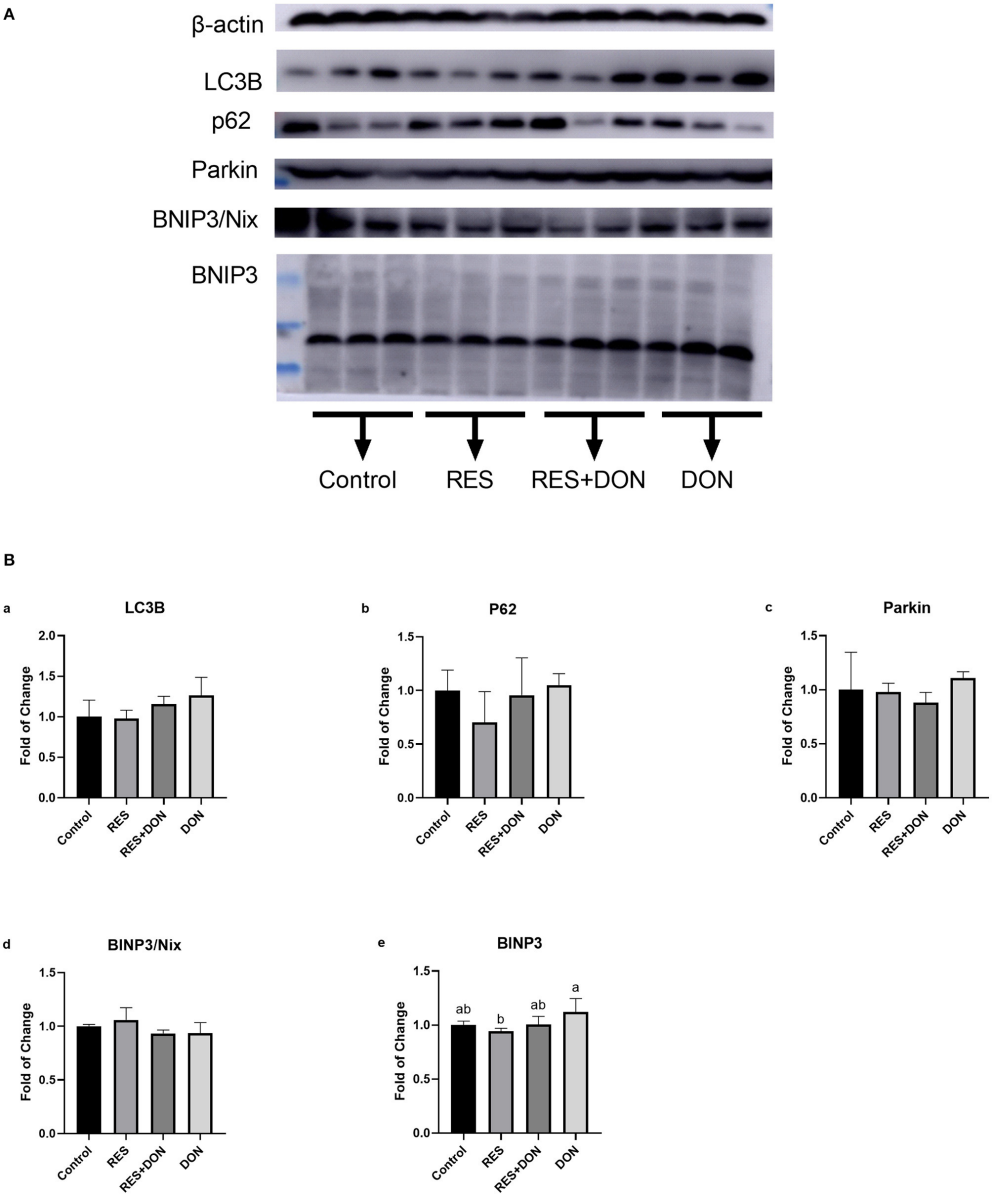
Dietary supplementation with RES increases the expression of mitophagy-related genes. Cells were treated with 0 (NC) or 0.5 μmol/L DON and 0 or 15 μM RES, respectively. Data were expressed as means ± SEM of at least three independent experiments. **(A,B)** Values with different letters are significantly different (*P* < 0.05).

Since we know that there are significant differences in mitophagy-related genes in the ileum, and then we analyzed the protein expressions of autophagy genes in ileum. The relative expression levels of LC3B, p62, Parkin, Binp3/Nix, and BINP3 are shown in Figure 2. Dietary supplementation with 3.8 mg DON/kg diet (DON) increased (*P* < 0.05) protein levels for BNIP3, while there were no differences (*P* > 0.05) in those indexes among the Control, RES, and RES+ DON treatments in the ileum.

### Dietary Supplementation With RES Affects the Expression of Mitochondrial Related Genes

**Figure 3:**
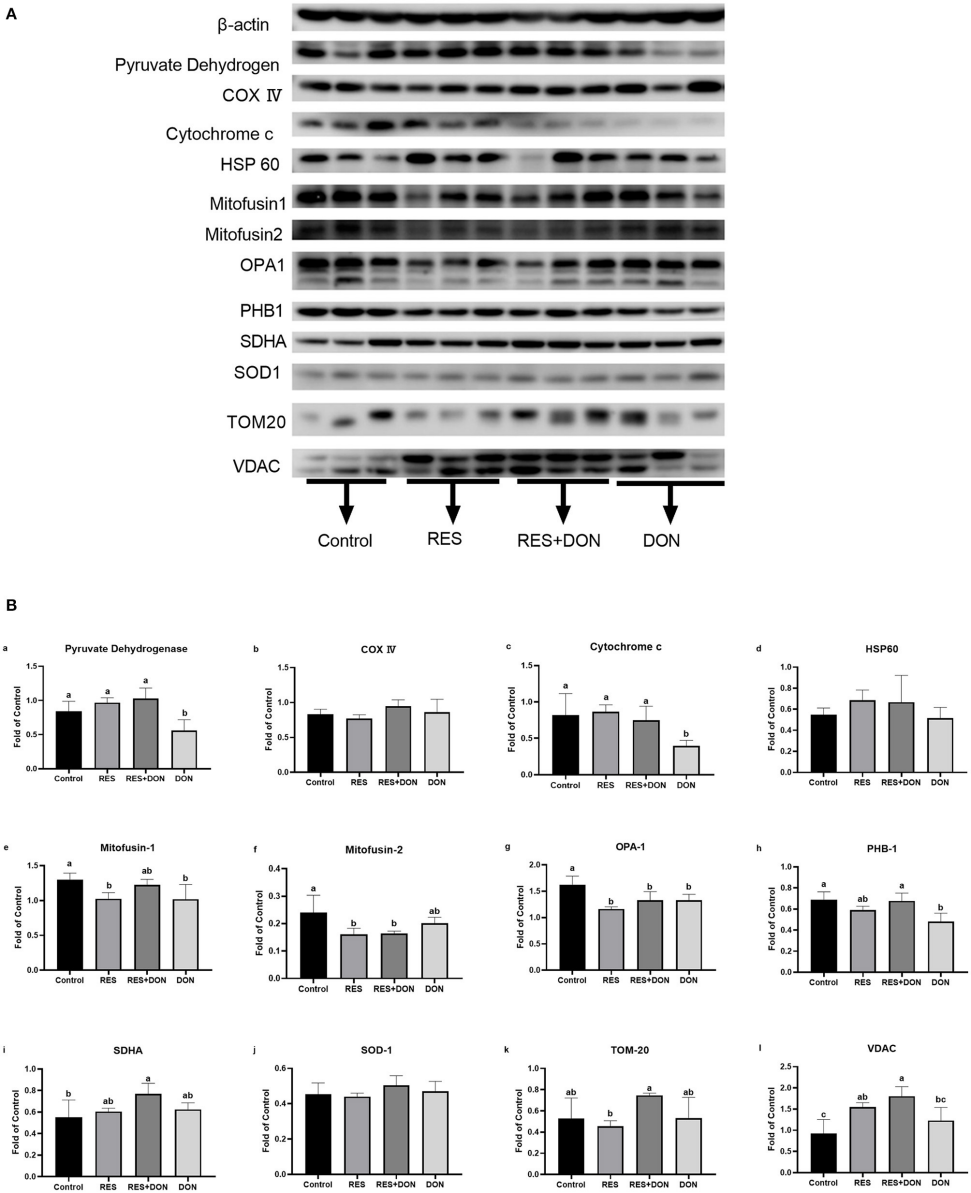
Dietary supplementation with RES affects the expression of mitochondrial related genes. Cells were treated with 0 (NC) or 0.5 μmol/L DON and 0 or 15 μM RES, respectively. Data were expressed as means ± SEM of at least three independent experiments. **(A,B)** Values with different letters are significantly different (*P* < 0.05).

In order to know whether the mitochondrial is related to this study, we analyzed the protein expressions of mitochondrial related genes in ileum. The relative expression levels of C54G1, COX IV, Cyt c, HSP60, Mitofusin1, Mitofusin2, OPA1, PHB1, SDHA, SOD1, TOM20, and VDAC are shown in Figure 3. Dietary supplementation with 3.8 mg DON/kg diet (DON) decreased (*P* < 0.05) protein levels for Pyruvate Dehydrogenase, Cytochrome c, MFN1, OPA1, and PHB1 (*P* < 0.05), while supplementation with 300 mg RES increased (*P* < 0.05) protein levels for PHB1, SDHA, and VDAC. Supplementation with RES decreased (*P* < 0.05) protein levels for MFN2 and OPA1, compared with control group.

### RES Improved the Negative Effect on Mitochondrial Respiration by DON *in vitro*

**Figure 4:**
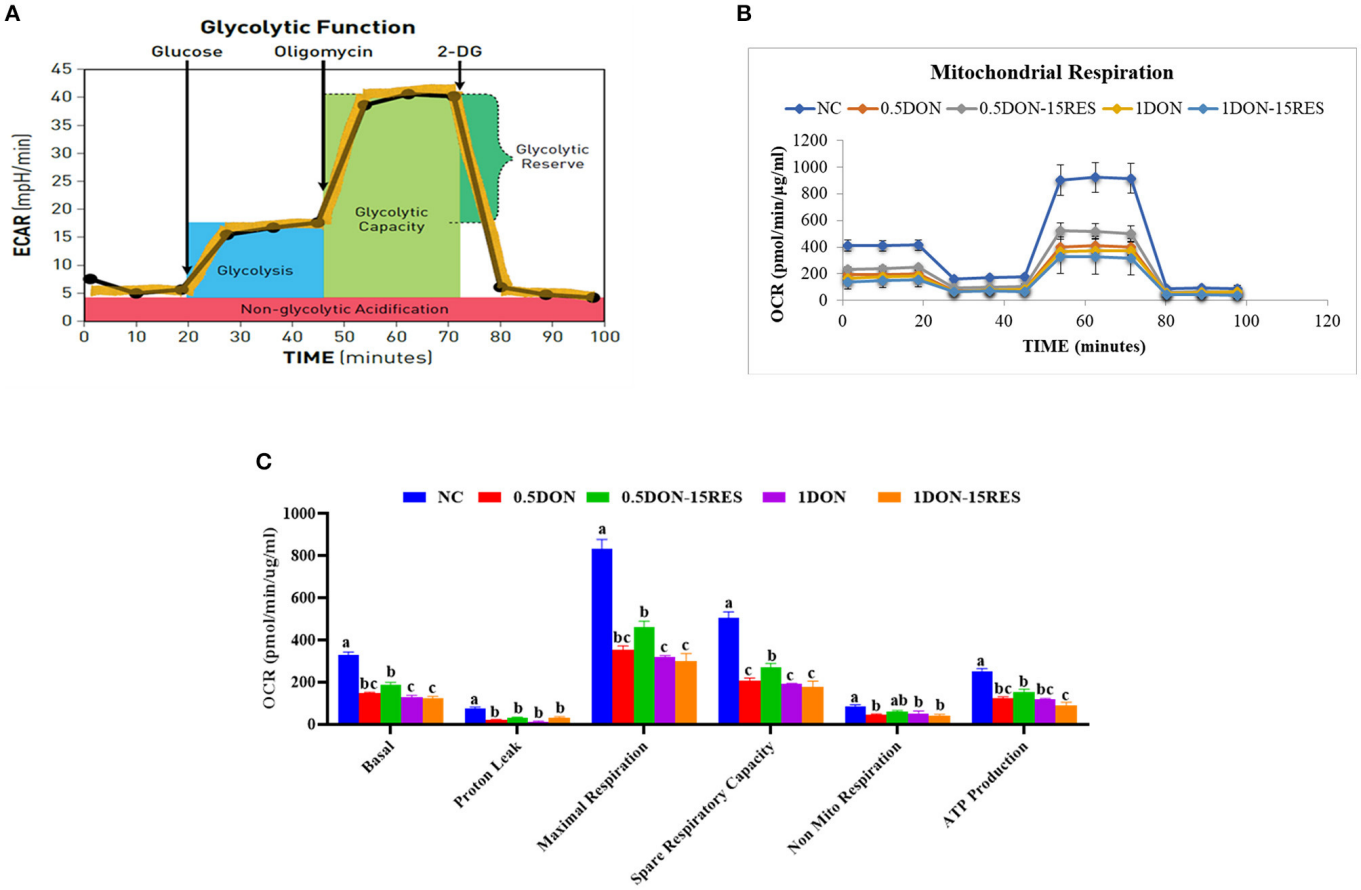
RES improved the negative effect on mitochondrial respiration by DON *in vitro*. **(A)** Schematic and **(B)** oxygen consumption rate (OCR) assessed by extracellular flux analysis. OCR was measured under basal conditions followed by the sequential addition of oligomycin (0.5 μM), FCCP (1 μM), rotenone (1 μM), or antimycin A (1 μM). Each data point represents an OCR measurement. **(C)** Individual parameters for basal respiration, proton leak, maximal respiration, spare respiratory capacity, nonmitochondrial respiration, and ATP production were determined. Cells were treated with 0 (NC) or 0.5μmol/L or 1μmol/L DON and 0 or 15μM RES, respectively. Data were expressed as means ± SEM of at least three independent experiments. **(A–C)** Values with different letters are significantly different (*P* < 0.05).

Our results have shown that RES ameliorates the damage challenged with DON through mitophagy, then we want to find out whether RES and DON affect the mitochondrial respiration *in vitro*. We found that supplementation with 0.5 μmol/L or 1 μmol/L DON gradually decreased (*P* < 0.05) individual parameters for basal respiration, proton leak, maximal respiration, spare respiratory capacity, non-mitochondrial respiration, and ATP production in cells. While supplementation with 15 μM RES elevated the rate of spare respiratory capacity in 0.5 μmol/L DON-treated cells (*P* < 0.05) but not normal cells (Figure 4).

**Figure 5:**
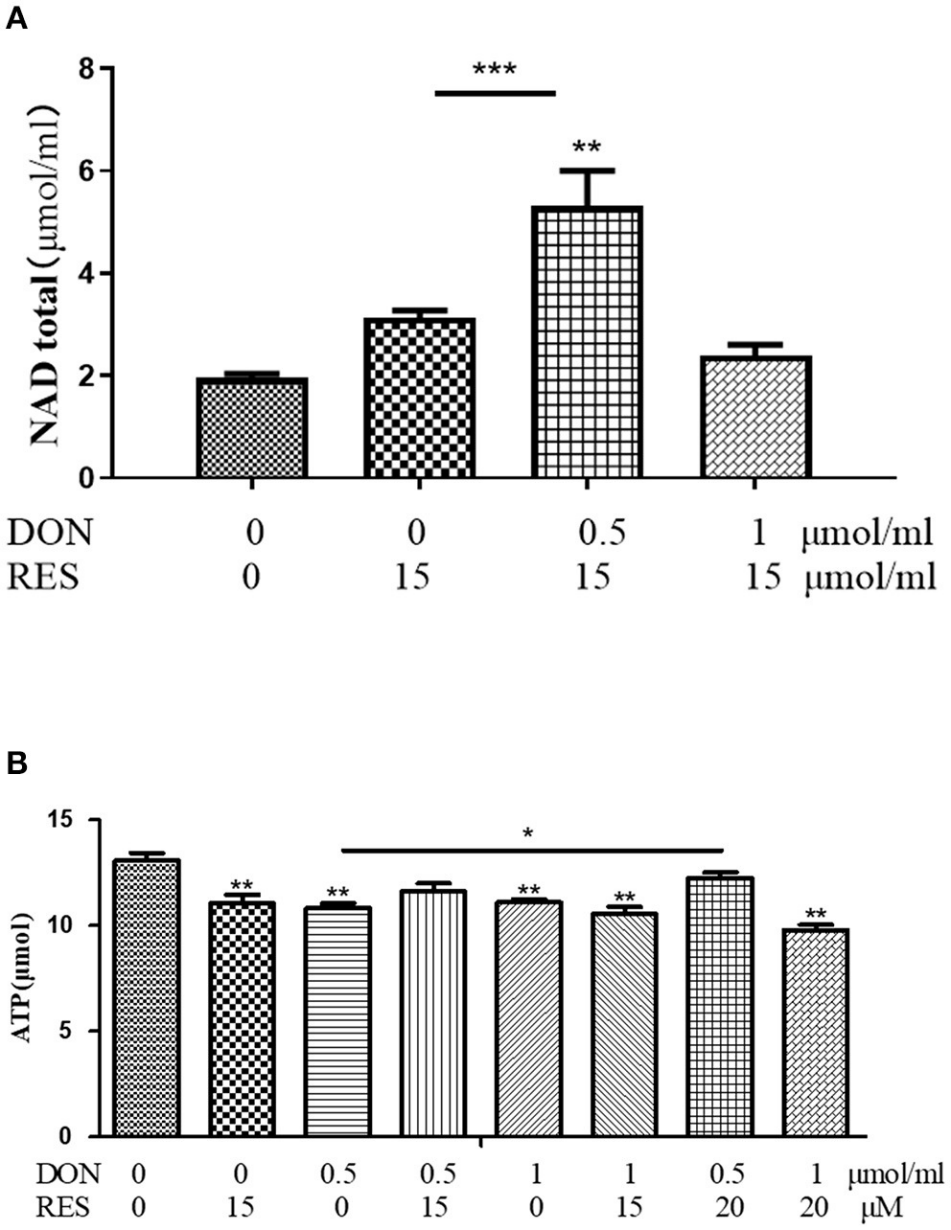
**(A,B)** Effect of DON and RES on Total NAD and ATP. Cells were treated with 0 (NC) or 0.5 μmol/L or 1 μmol/L DON and 0 or 15 μM or 20 μM RES, respectively. Data were expressed as means ± SEM of at least three independent experiments. *Values with different letters are significantly different (*P* < 0.05), *P < 0.05, **P < 0.01, ***P < 0.001.

The results of total NAD and ATP in IPEC-J2 cells are shown in Figure 5. supplementation with 0.5 μmol/L DON and 15 μM RES increased (*P* < 0.05) for total NAD, while supplementation with DON or RES alone decreased (*P* < 0.05) for ATP. However, addition of both 0.5 μmol/L DON and 20 μM RES increased (*P* < 0.05) the content of ATP.

### RES Did Not Relieve Injury on Mitochondrial Respiration Caused by DON When Knockout Atg5

**Figure 6:**
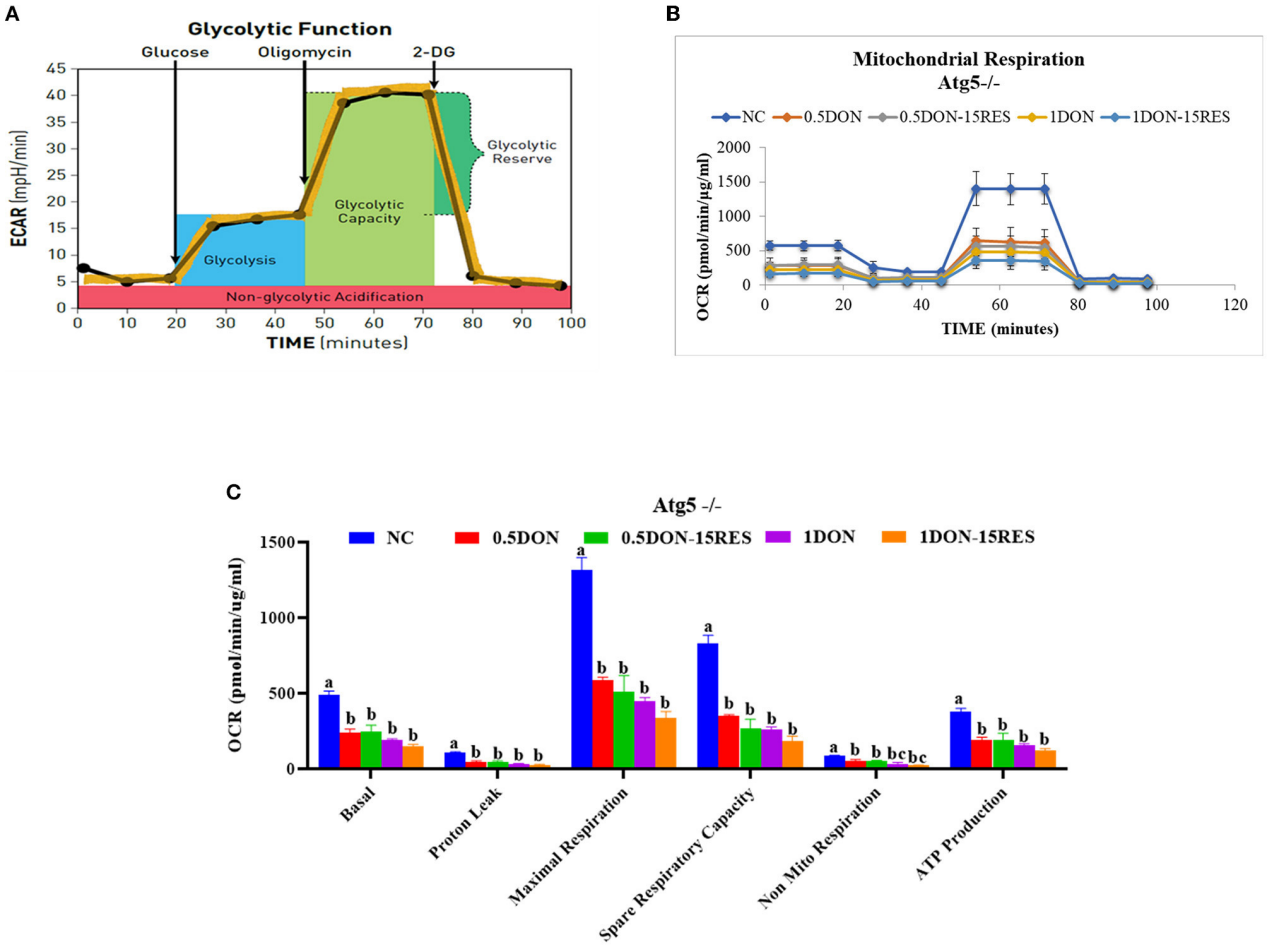
RES did not relieve injury on mitochondrial respiration caused by DON when knockout Atg5. **(A)** Schematic and **(B)** oxygen consumption rate (OCR) assessed by extracellular flux analysis. OCR was measured under basal conditions followed by the sequential addition of oligomycin (0.5 μM), FCCP (1 μM), rotenone (1 μM), or antimycin A (1 μM). Each data point represents an OCR measurement. **(C)** Individual parameters for basal respiration, proton leak, maximal respiration, spare respiratory capacity, nonmitochondrial respiration, and ATP production were determined. Cells were treated with 0 (NC) or 0.5 μmol/L or 1 μmol/L DON and 0 or 15 μM RES, respectively. Data were expressed as means ± SEM of at least three independent experiments. **(A–C)** Values with different letters are significantly different (*P* < 0.05).

The results of mitochondrial respiration in IPEC-J2 cells are shown in Figure 6. Supplementation with 0.5 μmol/L and 1 μmol/L DON gradually decreased (*P* < 0.05) individual parameters for basal respiration, proton leak, maximal respiration, spare respiratory capacity, non-mitochondrial respiration, and ATP production in cells. While supplementation with 15 μM RES there is no significant difference between the mitochondrial respiration.

## Discussion

DON is a common source of grain pollution, and has a negative impact on intestinal function and reduce growth performance for animals (14). Supplementation with 1 mg kg/DON damaged the intestinal morphology and impaired intestinal mucosa and permeability, accompanied by an inflammation response (15). 0.5 μg/ml DON cultured for 6 h in IPEC-J2 cells induced oxidative stress, inflammation and apoptosis. It has been reported that RES, as an effective antioxidant, can significantly increase cellular antioxidant enzyme activity, reduce intracellular ROS content, and decrease oxidative stress in intestinal epithelial cells, indicating that RES can be used as an effective feed additive to prevent intestinal damage in livestock production (16). Consistent with their research, the results of our study suggests that RES could improve the intestinal damage by DON. It shows RES plays an important role in protecting animal health.

RES regulates biological functions, such as anti-oxidative stress, anti-inflammatory, and antibacterial through molecular regulatory mechanisms (SIRT1, Nrf2, and NF-κB, etc). Moreover, RES is effective in preventing diseases like cardiovascular disease, diabetes, neurodegeneration, and cancer (17). In addition, RES can be used as an alternative to antibacterial feed additives to regulate piglets' intestinal flora, enhance antioxidant capacity in piglets' serum, reduce oxidative stress on piglets at weaning, and significantly improve growth performance of weaned piglets (18, 19). Recent study has shown that RES could be able to mitigate diquat-induced intestinal oxidative stress in piglets through mitochondrial autophagy (10). Interestingly, RES also enhances the transcription of BNIP3, a mitochondrial autophagy-related gene, through HIF1α and AMPK, thereby maintaining mitochondrial homeostasis and alleviating high-fat-induced endothelial function impairment (20).

VDAC is a class of pore-protein ion channels located in the outer mitochondrial membrane and plays a key role in regulating metabolism and energy fluxes across the outer mitochondrial membrane: it is involved in the transport of ATP, ADP, pyruvate, malate and other metabolites (21). VDAC expression was decreased in the DON group in our experiments, and there was an imbalance in the functions regulating mitochondrial outer membrane metabolism and energy. This situation was alleviated by the addition of RES. Cytochrome c plays a role in the electron transport chain and apoptosis, and also acts as an antioxidant enzyme in mitochondria to remove superoxide and hydrogen peroxide from mitochondria (22). Our results are consistent with previous studies, in that the expression of Cytochrome c protein was significantly reduced in the DON group, suggesting that the addition of DON caused oxidative damage to the cells. The damage was alleviated by the addition of RES. PHB1 has an important role in mitochondrial function and morphology and promotes cell proliferation in mice (23, 24). Consistent with the results in this experiment, PHB1 protein expression was decreased and cell proliferation was impaired in the DON group, and the addition of RES was associated with a recovery in PHB1 expression. All these proteins are closely associated with autophagy, and because the previous RT-PCR results showed that RES alleviates intestinal damage in relation to autophagy genes, we further validated the role of RES by testing the levels of these proteins. To sum up, our results have suggested that RES might alleviate DON-induced intestinal damage by improving mitochondrial autophagy.

Cellular respiration results in the conversion of nutrients into, for example, ATP, and then the release of a series of metabolic reaction products. In eukaryotic cells, mitochondria are important organelles for cellular respiration and are involved in the process of aerobic respiration. The nutrients protein, fat and carbohydrates in aerobic respiration are degraded by pyruvate to enter the tricarboxylic acid cycle to produce energy. In this paper, we examined the changes in the oxygen consumption rate of cells under RES treatment by Seahorse and found that 15 μM RES elevated the rate of spare respiratory capacity in 0.5 μmol/L DON-treated cells (*P* < 0.05) but not normal cells, however there is no significant change in basal respiration, proton leak, maximal respiration, non-mitochondrial respiration. When the autophagy-related gene ATG-5 was knocked out, there were no significant changes in the indicators of mitochondrial respiratory metabolism. In this experiment, the total intracellular ATP and NAD were also tested, and it was found that the addition of 0.5 μmol/L DON and 20 μM RES significantly increase for ATP, while addition of DON or RES alone significantly decrease. This indicates that RES could relieve DON-induced mitochondrial damage through mitophagy.

## Conclusions

RES alleviated the negative effects on mRNA and protein expression levels of mitophagy-related genes challenged with DON in piglets, elevated the rate of spare respiratory capacity, increased for ATP, and improved DON-induced mitochondrial damage *in vitro*. In conclusion, we have suggested that resveratrol would ameliorate the intestinal damage challenged with deoxynivalenol may through mitophagy in weaning piglets.

## Data Availability Statement

The raw data supporting the conclusions of this article will be made available by the authors, without undue reservation.

## Ethics Statement

The animal study was reviewed and approved by the Animal Care and Use Committee of Guangdong Academy of Agricultural Sciences and followed the Guidelines for the Care and Use of Animals for Research and Teaching. Written informed consent was obtained from the owners for the participation of their animals in this study.

## Author Contributions

YH and HX performed experiments, analyzed data, and wrote the manuscript. LW wrote and edited the manuscript. HX supervised the project, developed the study concept, and wrote and edited the manuscript. All authors contributed to the article and approved the submitted version.

## Funding

This study was jointly supported by the National Natural Science Foundation of China (31902172), Special fund for scientific innovation strategy-construction of high levelhigh-level Academy of Agriculture Science (R2020PY-JG009, R2017YJ-YB1004, R2018PY-JC001, and R2018PY-QF001), China Agriculture Research System of MOF and MARA; the Project of Swine Innovation Team in Guangdong Modern Agricultural Research System (2021KJ126), China.

## Conflict of Interest

The authors declare that the research was conducted in the absence of any commercial or financial relationships that could be construed as a potential conflict of interest.

## Publisher's Note

All claims expressed in this article are solely those of the authors and do not necessarily represent those of their affiliated organizations, or those of the publisher, the editors and the reviewers. Any product that may be evaluated in this article, or claim that may be made by its manufacturer, is not guaranteed or endorsed by the publisher.
